# Plasma membrane proteomic analysis of human Gastric Cancer tissues: revealing flotillin 1 as a marker for Gastric Cancer

**DOI:** 10.1186/s12885-015-1343-5

**Published:** 2015-05-07

**Authors:** Wen Gao, Jing Xu, Fuqiang Wang, Long Zhang, Rui Peng, Yongqian Shu, Jindao Wu, Qiyun Tang, Yunxia Zhu

**Affiliations:** Key Laboratory of Living Donor Liver Transplantation, Ministry of Public Health, Department of Liver Transplantation Center, The First Affiliated Hospital of Nanjing Medical University, 300 GuangZhou Road, Nanjing, 210029 China; Department of Gastroenterology, The first affiliated hospital of Nanjing medical university, 300 GuangZhou Road, Nanjing, 210029 China; Analysis Center of Nanjing Medical University, 104 Hanzhong Road, 210009 Nanjing, China; Department of Oncology, The first affiliated hospital of Nanjing medical university, 300 GuangZhou Road, Nanjing, 210029 China

**Keywords:** TMT, Gastric cancer, Plasma membrane, Flotillin 1, Biomarker

## Abstract

**Background:**

Gastric cancer remains the second leading cause of cancer-related deaths in the world. Successful early gastric cancer detection is hampered by lack of highly sensitive and specific biomarkers. Plasma membrane proteins participate and/or have a central role in the metastatic process of cancer cells and are potentially useful for cancer therapy due to easy accessibility of the targets.

**Methods:**

In the present research, TMT method followed by mass spectrometry analysis was used to compare the relative expression levels of plasma membrane proteins between noncancer and gastric cancer tissues.

**Results:**

Of a total data set that included 501 identified proteins, about 35% of the identified proteins were found to be plasma membrane and associated proteins. Among them, 82 proteins were at least 1.5-fold up- or down-regulated in gastric cancer compared with the adherent normal tissues.

**Conclusions:**

A number of markers (e.g. annexin A6, caveolin 1, epidermal growth factor receptor, integrin beta 4) were previously reported as biomarkers of GC. Additionally, several potential biomarkers participated in endocytosis pathway and integrin signaling pathways were firstly identified as differentially expressed proteins in GC samples. Our findings also supported the notion that flotillin 1 is a potential biomarker that could be exploited for molecular imaging-based detection of gastric cancer. Together, the results show that subcellular proteomics of tumor tissue is a feasible and promising avenue for exploring oncogenesis.

**Electronic supplementary material:**

The online version of this article (doi:10.1186/s12885-015-1343-5) contains supplementary material, which is available to authorized users.

## Background

Gastric cancer(GC) is the second leading cause of cancer related deaths which kill about 800 000 people annually [1]. It is a highly aggressive malignant disease with the overall 5 year survival rate (5YSR) of 24% [2]. The major reason for this poor outcome is the difficulty in the detection of early stage GC when treatment could improve long term survival of patients. Therefore, the identification of tumor biomarkers for early detection plays an important role in improving diagnosis and treatment of GC. Unfortunately, tumor biomarkers such as CEA and CA19-9 that are currently utilized for the detection of GC in clinical practice are not specific and sensitive enough; with their sensitivity in the range of 18%–57% [3]. Consequently, discovery of the valuable biomarkers of GC remains a worthy task.

Plasma membrane encloses the cell and maintains the essential boundaries between the cytosol and the extracellular environment. The proteins constitute approximately 50% (by mass) of the cell surface membrane [4]. Proteins located in plasma membrane mediate most functions of the membrane, such as acting as sensors for external signals, transporters of specific molecules and the connection point of the membrane to the cytoskeleton, the extracellular matrix and adjacent cells [5,6]. Significantly, these proteins constitute more than 45% of current drug targets, with 25–30% of drugs targeting G-protein coupled receptors [7,8]. Defining the plasma membrane proteome is of great interest due to the fundamental role of membrane proteins [9]. Moreover, profiling plasma membrane markers in specific disease stage has great potential for identifying novel biomarkers and subsequent therapeutic targets [8]. Her2 [10], c-Met, and EGFR [11] are classical examples of plasma membrane proteins against which small molecules and biologics have been successfully developed and implemented in the clinic [10-12]. Attempts have succeeded in identifying potential plasma membrane biomarkers of GC from cell lines. These include but are not limited to SLC3A2. However, global proteomic analysis of membrane-enriched samples from normal versus GC tissues has not been reported before.

Stable isotope-based quantitative proteomics approach for identification and quantification of proteins has provided new possibilities in the field of biomarker discovery [13]. The isotopes can be incorporated metabolically as in SILAC or chemically as in isobaric labeling methods include isobaric tag for relative and absolute quantification (iTRAQ) and tandem mass tag (TMT) [14,15]. The 2-plex and 6-plex tandem mass tags (TMTs), through the incorporation of, respectively, one (^13^ C) and five (^13^C or ^15^ N) stable isotopes, perform relative protein quantification between two and up to six samples [16]. This method is successfully used to screen for biomarkers in periodontal disease, colorectal cancer [15], breast cancerand so on [17,18].

In this study, we used TMT label combined with LC-MS/MS to compare the expression level of plasma membrane proteins between a pair of “normal” and gastric cancer tissues, thereby allowing identification of plasma membrane-associated biomarkers. Our data revealed flotillin 1 plasma membrane protein to be a potential biomarker for GC detection.

## Methods

### Patient samples

GC samples with stage I tumors((AJCC 6th Edition Stage I disease, with minimal depth of invasion into mucosa and no metastatic lymph nodes) and matched normal tissue samples (50–200 mg) were obtained from surgical resection specimens at the department of pathology, snap frozen in liquid nitrogen, and stored at -80°C until use and subjected to routine pathological examination at Jiangsu province hospital. The patients’ age ranged from 32 to 90 years, only 12 patients were GC and were available for further studies. Written informed consent was obtained from each patient before surgery. This study was approved by the Ethics Committee of Nanjing Medical University with an Institutional Review Board (IRB) number of 2012-NFLZ-32. The tumor and control samples were pooled separately and subjected to proteomic analysis.

### Plasma membrane purification and protein lysis

Plasma membrane was enriched as previously described [19]. Briefly, tissues were lysed by hypotonic buffer (10 mM Trisbase, 1.5 mM MgCl_2_, 10 mM NaCl, pH 6.8) for 5 min followed by centrifugation at 300 × *g* for 5 min, then resuspended in gradient buffer (0.25 M Sucrose, 10 mM HEPES, 100 mM Succinic acid, 1 mM EDTA, 2 mM CaCl_2_, 2 mM MgCl_2_, pH7.4) and homogenized. The homogenate was centrifuged at 1,000 × *g* for 10 min and the supernatant was collected. Subsequently, the supernatant was centrifuged at 100 000 × *g* for 30 min. The pellet was purified membranes which were resuspended in 2 mL gradient buffer by homogenization and mixed with 1.9 mL Percoll (Amersham Biosciences, Uppsala,Sweden) containing 10% PBS and 0.19 mL 2.5 M sucrose in an Easy-Seal tube (polyallomer, 5 mL, Sorvall). The tube was filled with gradient buffer, capped and centrifuged at 120 000 × *g* for 15 min. The pellet was washed with ice-cold PBS three times and suspended in 150 μl of SDS lysis buffer and stored at −80°C. The protein concentrations were determined by the Bradford method.

### Protein digestion and peptide tandem mass tag(TMT) labeling

Protein digestion and TMT labeling were done as previously described [20]. 1 mg of plasma membrane protein from normal or GC samples was reduced with 10 mM DTT at 60°C for 1 h, alkylated with 55 mM IAA for 45 min at room temperature in the dark and digestion with trypsin overnight at 37°C. Tryptic peptides were desalted and then dried in vacuo (Speed Vac, Eppendorf). 20 μg of proteins was labeled for 1 h at room temperature by adding 5 μL of the TMT reagent. The peptides were labeled with isobaric tags and mixed at 1:1 ratio based on total peptide amount. The TMT labeled proteins were stored at -80°C until used.

### SCX fractionation separation

SCX fractionation separation was done as previously described [20]. Peptide mixtures were resuspended in 10 mM NH_4_COOH, 5% ACN( pH 2.7), and subjected to cation ion exchange columns (1 mm ID × 10 cm packed with Poros 10 S, DIONEX, Sunnyvale,CA, USA) with the UltiMate® 3000 HPLC system. The separation was performed by applying a two-buffer system. Buffers A and B were prepared as follows: buffer A, 5 mM ammonium formate, 5% ACN (pH = 2.7); buffer B, 800 mM ammonium formate, 5% ACN (pH = 2.7).The following gradient was employed: 0% to 30% B for 21 min, 30% to 56% B for 7 min, 56% B to 100% B for 1 min, 100% B for 3 min, 100% B to 0% for 1 min and 0% for 20 min before the next run. Twenty fractions in total were collected and lyophilized.

### Mass spectrometry analysis

Mass spectrometry analysis was done as previously described [21]. The labeled peptides were analyzed on the LTQ Orbitrap-Velos instrument (Thermo Fisher, USA) connecting to a Nano ACQUITY UPLC system via a nanospray source. The reverse-phase separation of peptides was performed using the buffer A(2% ACN, 0.5% acetic acid) and buffer B (80% ACN, 0.5% acetic acid); the gradient was set as following: 4% to 9% buffer B for 3 min, 9% to 33% buffer B for 170 min, 33% to 50% buffer B for 10 min, 50% to 100% buffer B for 1 min, 100% buffer B for 8 min, 100% to 4% buffer B for 1 min. For analysis of plasma membrane proteins, one full scan was followed by the selection of the eight most intense ions for collision-induced dissociation (CID) fragmentation (collision energy 35%). The most intense product ion from the MS2 step was selected for higher energy collision-induced dissociation (HCD)-MS3 fragmentation.

### Protein identification and quantification

Protein identification and quantification were done as previously described [21]. Maxquant (version 1.2.2.5) was used to identify the raw spectra acquired from precursor ions as described [22]. Search parameters were set as following: precursor mass tolerance of ± 20 parts per million (ppm); 0.5-dalton product ion mass tolerance; trypsin digestion; up to two missed cleavages; carbamidomethylation (+57.02146 Da) on cysteine, TMT reagent adducts (+229.162932 Da) on lysine and peptide amino termini were set as a fixed modification; and methionine oxidation (+15.99492 Da) was set as a variable modification. False discovery rates (FDR) of the identified peptides and proteins were estimated by searching against the database with the reversed amino acid sequence. Only peptides with at least six amino acids in length and an FDR of 1% were considered to be successfully identified. Relative protein abundance ratios between two groups were calculated from TMT reagent reporter ion intensities from HCD spectra. For TMT labeling, each peptide channel was re-normalized by the sum across channels. The protein intensity was calculated as the median of normalized intensity of the corresponding peptides. The mean and standard deviation for each protein across subjects was calculated, and Perseus was used to perform statistical comparisons. One-way analysis of variance (ANOVA) was used to calculate significant differences in abundance among groups. A permutation-based FDR value less than 0.05 was considered significant.

### Ingenuity pathway analysis

To further explore the significance of the differentially expressed plasma membrane proteins, Ingenuity® Pathway Analysis (IPA; Ingenuity® Systems, www.ingenuity.com/) was used to search the relevant molecular functions, cellular processes and pathways of these identified proteins during the pathological changes of GC. Associated networks of differentially expressed plasma membrane proteins were generated, along with a score representing the log probability of a particular network being found by random chance. Top canonical pathways associated with the uploaded data were presented, along with a p-value. The p-values were calculated using right-tailed Fisher’s exact tests.

### Western blot analyses

Lysates from normal or GC plasma membrane samples were separated on 11.5% SDS-PAGE gels and then the proteins were transferred to nitrocellulose membranes, blocked in TBST containing 5% nonfat milk powder for 4 hour and incubated overnight with primary antibodies against Na^+^/K^+^-ATPase(Abcam Ab76020, 1:1000), prohibitin(Abcam Ab28172, 1:1000), Golgi 58(Abcam Ab27043, 1:500), histone H2A(Abcam Ab18255, 1:1000), sigma non-opioid intracellular receptor 1(Abcam Ab160924, Cambridge, UK; 1:1000), flotillin 1(Abcam Ab41927, 1:500), CD36 (Abcam Ab78054, 1:500) and CD9 molecule (Abcam Ab65230, 1:500), then washed three times with TBST. The membranes were incubated for 1 hour with alkaline phosphatase (AP)-conjugated anti-mouse or rabbit IgG. The protein levels were evaluated by the detection of activity of alkaline phosphatase using a Lumi-Phos kit (Pierce Biotechnology). The visualized bands of western blot were quantified with Bio-Rad QUANTITY ONE software. The volumes of target bands were normalized to GAPDH. The average absolute intensity and the standard deviation were determined. The protein ratio was determined using these averaged values. Student’s T-test was used to generate p values. Significant difference was recognized as a p value less than 0.05.

### Immunohistochemistry and tissue microarray

For expression studies of human flotillin 1 in clinical samples, we used tissue microarrays purchased from Biomax, Inc. [ST1004 and bST801a)] containing cores from a total of 85 different cases of GC with matched adjacent normal tissues and an additional 10 normal only tissues. IHC of tissue arrays was done as described previously. Flotillin 1 protein expression was assessed using a previously described semiquantitative scoring consisting of an assessment of both staining intensity (scale 0 to 3) and the percentage of positive cells (0 to 100%), which, when multiplied, generate a score ranging from 0 to 300. Statistical analysis was done using SPSS 18.0. The t test was performed at 95% confidence.

## Results

### Detection of plasma membrane proteins in GC and adjacent normal tissues

**Figure 1:**
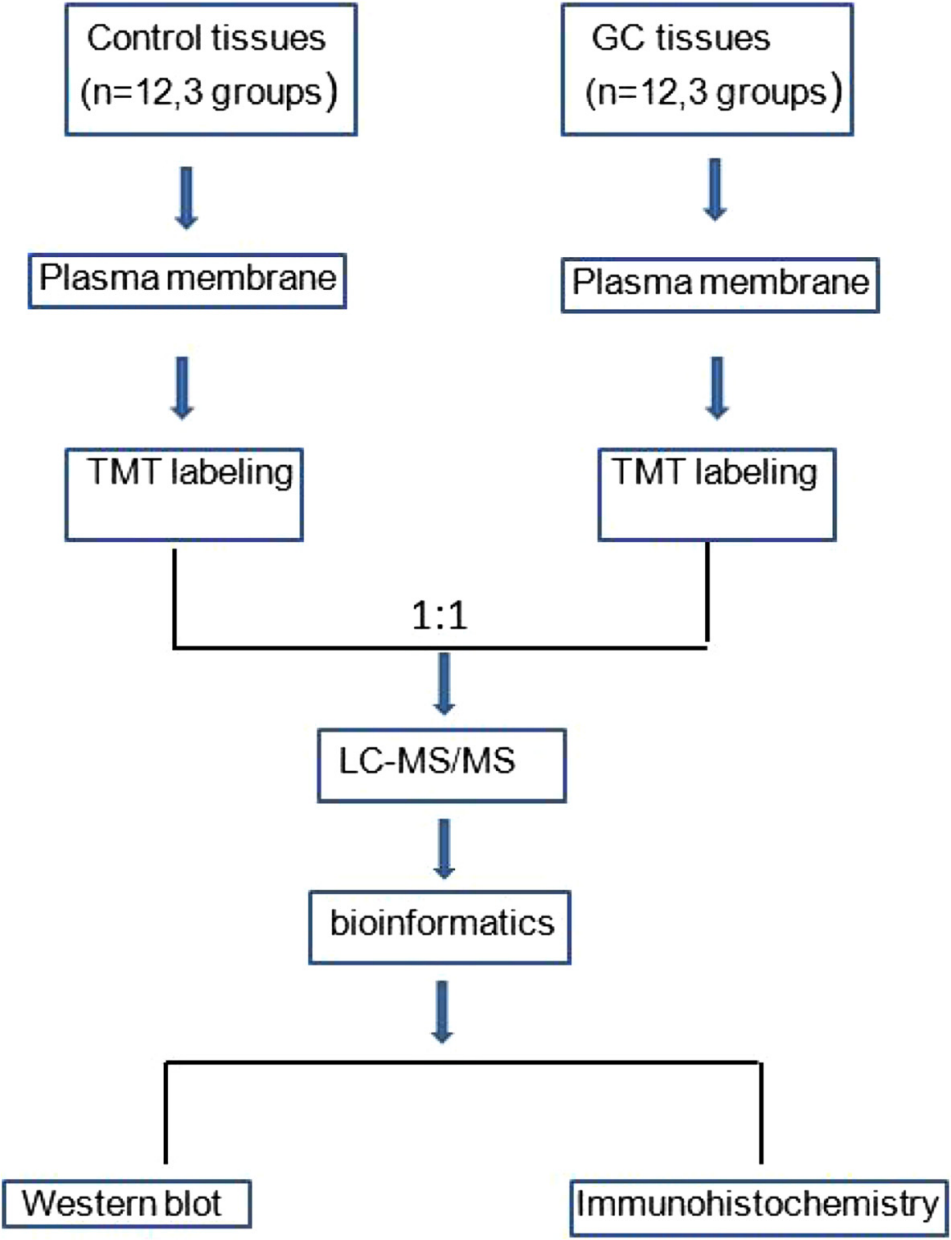
Schematic representation of the strategy used to identify the differentially expressed proteins in GC tissues.

**Figure 2:**
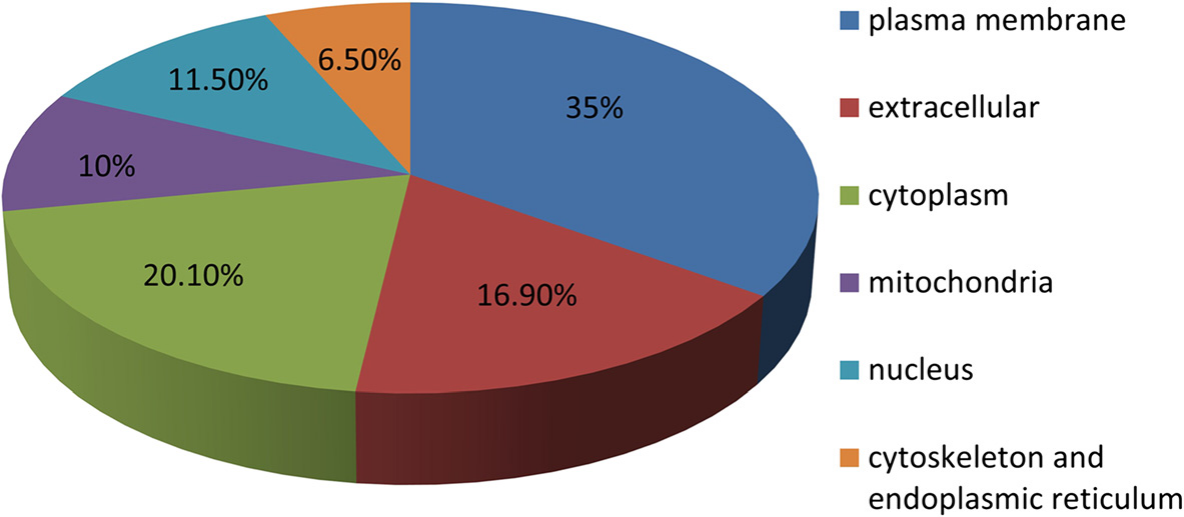
Western blot analysis of the plasma membrane from GC and control tissues after Percoll density gradient centrifugation; The same amount of proteins (50 *μ*g) was loaded on each lane.

**Figure 3:**
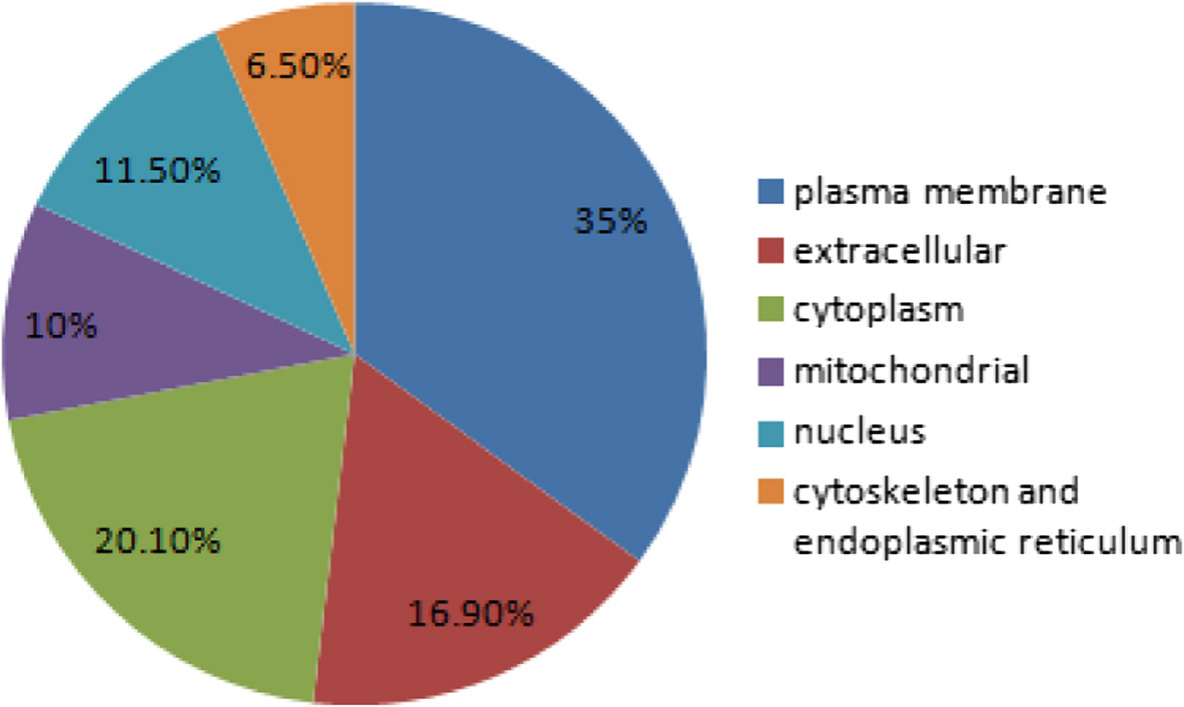
The subcellular locations of the identified proteins from GC and normal tissues according to the GO annotations and literature.

The experimental workflow of this study is shown in Figure 1. To discover plasma membrane protein alterations associated with GC, six pools of plasma membrane samples (three controls and three GC) were generated by pooling samples from 4 subjects for each pool. The purity of the plasma membrane after Percoll density gradient centrifugation was detected by western blot analysis. Figure 2 indicated that the plasma membrane was highly enriched in the marker, Na^+^/K^+^-ATPase, compared to the total lysis fraction. A total of 501 proteins were identified in the workflow (Additional file 1: Table S1). To further assess the efficacy of the protocol for the enrichment of plasma membrane proteins, the subcellular locations and functions were cataloged according to the gene ontology (GO) component annotations from literatures. Figure 3 showed that 175 proteins (about 35%) have been assigned as plasma membrane or membrane-associated proteins. Of the remaining proteins with subcellular annotation, approximately 16.9% of the identified proteins are extracellular, and 20.1% proteins locate in cytoplasm. 10% proteins locate in mitochondria and 11.5% proteins are nuclear or nuclear associated proteins. Other 6.5% proteins are mainly from cytoskeleton and endoplasmic reticulum.

### Quantification of plasma membrane proteins in GC and adjacent normal tissues

**Figure 4:**
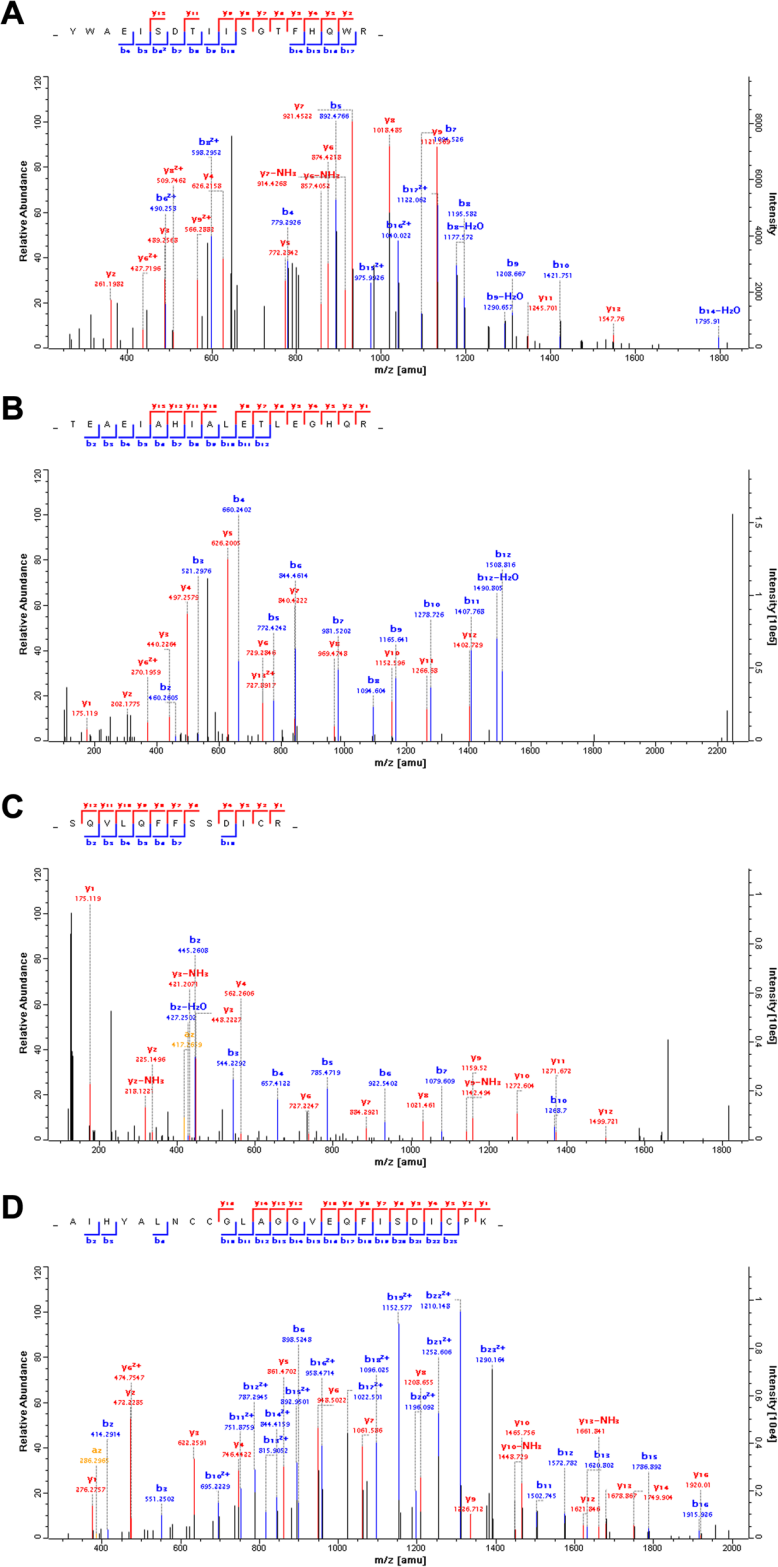
Mass spectra of four representative proteins. **(A)** sigma non-opioid intracellular receptor 1, **(B)** flotillin 1, **(C)** CD 36 and **(D)** CD9.

Proteins were labeled with TMT reagents and analyzed using tandem mass spectrometry to screen for the differentially expressed proteins between GC and adjacent normal tissues. To increase the coverage of protein identifications and the confidence of the data generated, three pools of adjacent normal tissues were labeled with TMT reagents 126, 127 and 128 respectively; pools of GC tissues were labeled with TMT reagents 129, 130 and 131 respectively. The relative quantification analysis by Maxquant 1.2.2.5 software comes with statistical analysis, however, most methods are prone to technical variation, so we included an additional 1.5-fold cut off for all TMT ratios to add stringency when classifying proteins as up- or down-regulated. A total of 205 differentially expressed proteins proteins were identified with 95% confidence (Additional file 2: Table S2). Of these, 82 plasma membrane proteins were found to have >1.5-fold difference in expression between the GC and adjacent normal tissues (Table 1). 24 proteins were downregulated in gastric cancer, whereas 58 were overexpressed compared to adjacent normal tissues. The plasma membrane/plasma membrane -associated proteins comprised about 40% of the total proteins detected. The mass spectra of four representative proteins (sigma non-opioid intracellular receptor 1, flotillin 1, CD36 and CD9 molecule) were shown in Figure 4.

**Table 1:** **Differentially regulated plasma membrane proteins identified in GC tissues**

**Accession No.**	**Gene symbols**	**Description**	**TMT ratio**	**P value**	**Function**
Q12959	DLG1	discs, large homolog 1 (Drosophila)	-8.78	1.28E-05	kinase
G8JLH6	CD9	CD9 molecule	-3.723	7.04E-02	other
P30453	HLA-A	major histocompatibility complex, class I, A	-2.903	7.40E-04	other
O95810	SDPR	serum deprivation response	-2.623	1.69E-09	other
P48960	CD97	CD97 molecule	-2.615	3.74E-01	G-protein coupled receptor
Q16853	AOC3	amine oxidase, copper containing 3	-2.314	1.11E-10	enzyme
E7EWP3	MPZ	myelin protein zero	-2.266	2.45E-07	other
Q03135	CAV1	caveolin 1, caveolae protein, 22 kDa	-2.251	2.43E-10	transmembrane receptor
P41219	PRPH	peripherin	-2.149	1.65E-08	other
P43121	MCAM	melanoma cell adhesion molecule	-2.115	1.93E-09	other
Q9HAV0	GNB4	guanine nucleotide binding protein (G protein), beta polypeptide 4	-1.976	9.69E-04	enzyme
Q86UP2	KTN1	kinectin 1 (kinesin receptor)	-1.959	7.69E-03	transmembrane receptor
P01859	IGHG2	immunoglobulin heavy constant gamma 2 (G2m marker)	-1.844	1.43E-02	other
B7Z2R4	SGCE	sarcoglycan, epsilon	-1.791	2.91E-02	other
P18206	VCL	vinculin	-1.685	2.01E-10	enzyme
A0FGR8	ESYT2	extended synaptotagmin-like protein 2	-1.672	2.68E-03	other
P08133	ANXA6	annexin A6	-1.668	1.10E-11	ion channel
O43491	EPB41L2	erythrocyte membrane protein band 4.1-like 2	-1.66	1.05E-08	other
Q14BN4	SLMAP	sarcolemma associated protein	-1.645	6.33E-04	other
A6NMH8	CD81	CD81 molecule	-1.634	3.17E-04	other
Q96CX2	KCTD12	potassium channel tetramerization domain containing 12	-1.559	3.59E-08	ion channel
Q9H223	EHD4	EH-domain containing 4	-1.527	1.30E-03	enzyme
Q13418	ILK	integrin-linked kinase	-1.526	1.20E-09	kinase
P16671	CD36	CD36 molecule (thrombospondin receptor)	-1.501	1.16E-06	transmembrane receptor
P38606	ATP6V1A	ATPase, H+ transporting, lysosomal 70 kDa, V1 subunit A	1.514	3.79E-05	transporter
Q14108	SCARB2	scavenger receptor class B, member 2	1.514	1.92E-08	other
P01903	HLA-DRA	major histocompatibility complex, class II, DR alpha	1.521	2.94E-08	transmembrane receptor
Q14444	CAPRIN1	cell cycle associated protein 1	1.529	1.30E-06	other
Q6IAA8	LAMTOR1	late endosomal/lysosomal adaptor, MAPK and MTOR activator 1	1.533	5.18E-03	other
P27216	ANXA13	annexin A13	1.536	3.38E-09	other
Q9Y287	ITM2B	integral membrane protein 2B	1.553	7.16E-02	other
Q9Y6R1	SLC4A4	solute carrier family 4 (sodium bicarbonate cotransporter), member 4	1.558	2.21E-04	transporter
Q8WVV4	POF1B	premature ovarian failure, 1B	1.568	9.94E-09	other
D6RH31	NPNT	nephronectin	1.59	1.40E-05	other
Q9P0L0	VAPA	VAMP (vesicle-associated membrane protein)-associated protein A, 33 kDa	1.597	7.50E-06	other
P02786	TFRC	transferrin receptor	1.602	3.53E-01	transporter
O00203	AP3B1	adaptor-related protein complex 3, beta 1 subunit	1.619	5.16E-03	transporter
Q00610	CLTC	clathrin, heavy chain (Hc)	1.623	6.18E-11	other
P26006	ITGA3	integrin, alpha 3 (antigen CD49C, alpha 3 subunit of VLA-3 receptor)	1.645	3.24E-08	other
O95292	VAPB	VAMP (vesicle-associated membrane protein)-associated protein B and C	1.652	4.17E-05	other
B4DNJ6	STRAP	serine/threonine kinase receptor associated protein	1.654	4.56E-03	other
B5MCA4	EPCAM	epithelial cell adhesion molecule	1.655	4.47E-03	other
Q14247	CTTN	cortactin	1.664	2.58E-06	other
C9JME2	FARP1	FERM, RhoGEF (ARHGEF) and pleckstrin domain protein 1 (chondrocyte-derived)	1.667	4.07E-10	other
P15144	ANPEP	alanyl (membrane) aminopeptidase	1.693	7.85E-10	peptidase
G5EA09	SDCBP	syndecan binding protein (syntenin)	1.71	1.01E-06	enzyme
O75955	FLOT1	flotillin 1	1.727	2.09E-02	other
Q86XK7	VSIG1	V-set and immunoglobulin domain containing 1	1.727	1.27E-04	other
P00533	EGFR	epidermal growth factor receptor	1.755	2.27E-01	kinase
P17301	ITGA2	integrin, alpha 2 (CD49B, alpha 2 subunit of VLA-2 receptor)	1.786	1.19E-08	transmembrane receptor
P29992	GNA11	guanine nucleotide binding protein (G protein), alpha 11 (Gq class)	1.786	7.56E-02	enzyme
C9J6P4	ZC3HAV1	zinc finger CCCH-type, antiviral 1	1.835	3.35E-09	other
P27105	STOM	stomatin	1.844	1.31E-04	other
P46977	STT3A	STT3A, subunit of the oligosaccharyltransferase complex (catalytic)	1.895	5.89E-05	enzyme
Q9BX66	SORBS1	sorbin and SH3 domain containing 1	1.895	1.62E-08	other
O95563	MPC2	mitochondrial pyruvate carrier 2	1.917	2.32E-08	other
P09496	CLTA	clathrin, light chain A	1.926	1.13E-12	other
Q13155	AIMP2	aminoacyl tRNA synthetase complex-interacting multifunctional protein 2	1.928	1.03E-07	other
P09497	CLTB	clathrin, light chain B	1.954	1.00E-10	other
H9KV28	DIAPH1	diaphanous-related formin 1	1.958	8.62E-04	other
Q99720	SIGMAR1	sigma non-opioid intracellular receptor 1	1.961	3.62E-04	G-protein coupled receptor
P13473	LAMP2	lysosomal-associated membrane protein 2	1.999	5.05E-10	enzyme
F5H7K4	NCEH1	neutral cholesterol ester hydrolase 1	2.006	5.68E-04	enzyme
P11215	ITGAM	integrin, alpha M (complement component 3 receptor 3 subunit)	2.012	2.29E-10	transmembrane receptor
P15924	DSP	desmoplakin	2.031	8.86E-13	other
Q9UGM3	DMBT1	deleted in malignant brain tumors 1	2.079	2.59E-06	transmembrane receptor
Q93008	USP9X	ubiquitin specific peptidase 9, X-linked	2.19	1.22E-01	peptidase
O15400	STX7	syntaxin 7	2.208	1.94E-07	transporter
Q9BXJ0	C1QTNF5	C1q and tumor necrosis factor related protein 5	2.223	2.33E-04	transmembrane receptor
Q9UJZ1	STOML2	stomatin (EPB72)-like 2	2.228	1.06E-04	other
P55011	SLC12A2	solute carrier family 12 (sodium/potassium/chloride transporter), member 2	2.332	5.91E-11	transporter
O00182	LGALS9	lectin, galactoside-binding, soluble, 9	2.45	1.39E-05	other
Q08380	LGALS3BP	lectin, galactoside-binding, soluble, 3 binding protein	2.469	4.66E-06	transmembrane receptor
Q9UGI8	TES	testis derived transcript (3 LIM domains)	2.577	1.55E-06	other
Q9NZ01	TECR	trans-2,3-enoyl-CoA reductase	2.83	1.45E-07	enzyme
G3V1K3	PON2	paraoxonase 2	2.932	3.93E-06	enzyme
O95841	ANGPTL1	angiopoietin-like 1	3.109	4.33E-04	other
Q92542	NCSTN	nicastrin	3.634	3.58E-02	peptidase
O95497	VNN1	vanin 1	3.718	1.00E-08	enzyme
P16144	ITGB4	integrin, beta 4	3.792	2.40E-01	transmembrane receptor
Q96HR9	REEP6	receptor accessory protein 6	3.797	2.70E-08	other
P48230	TM4SF4	transmembrane 4 L six family member 4	4.71	1.42E-04	other

### Functional characteristics of the proteins detected in GC and adjacent normal tissues

**Figure 5:**
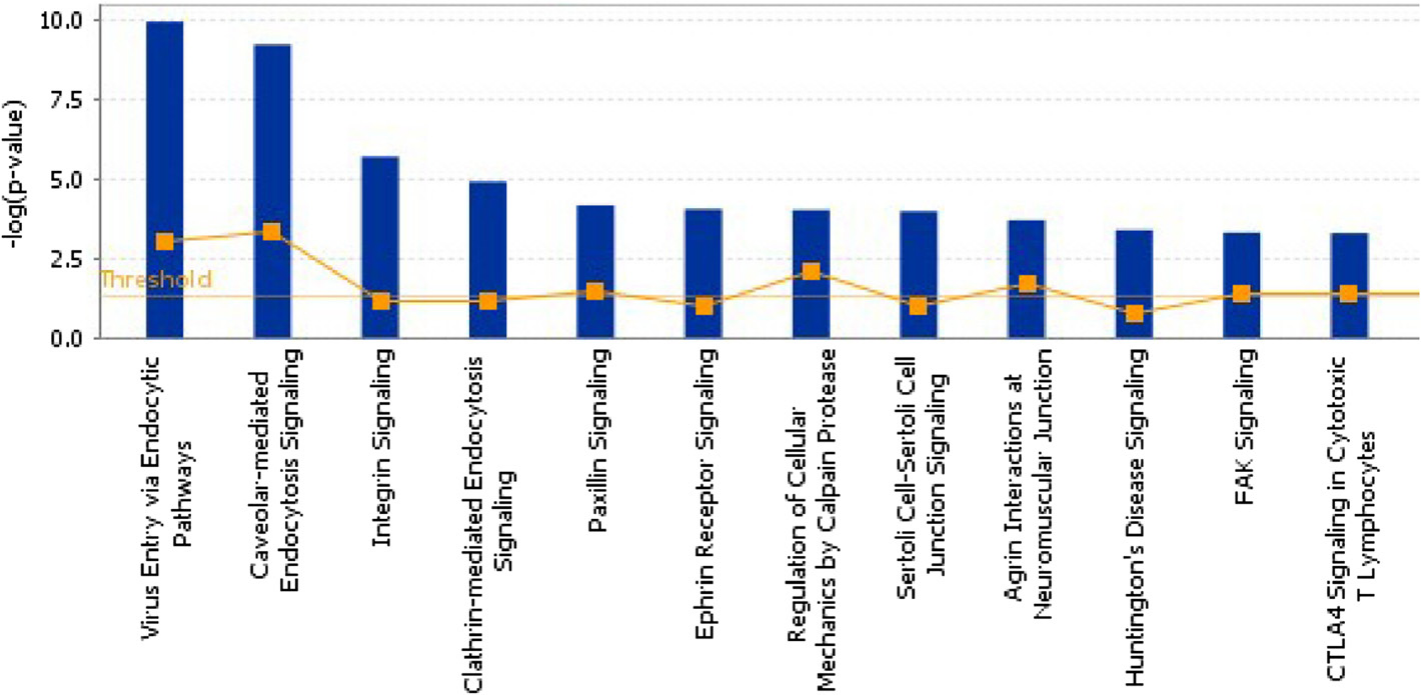
Ingenuity Pathway Analysis of proteins that were significantly altered in pathways.

To better appreciate the molecular and functional characteristics of the 82 differentially expressed plasma membrane or membrane-associated proteins, these proteins were subjected to IPA analysis for further identification of important biological processes that they were significantly involved in. The over-represented biological processes, molecular functions, and canonical pathways were generated based on information contained in the Ingenuity Pathways Knowledge Base. We found that the top three significant biological processes of the differentially expressed proteins in our study were networks describing 1) cancer, renal and urological system development and function, tissue morphology; 2) cell-to-cell signaling and interaction, infectious disease, cellular function and maintenance; 3) cellular assembly and organization, nervous system development and function, cellular movement. For molecular and cellular functions, the data indicated that many proteins involved in cellular function and maintenance, cell-to-cell signaling and interaction, cell morphology and cellular movement. Our results showed that the top three canonical pathways of differentially expressed proteins participated in were virus entry via endocytic pathways, caveolar-mediated endocytosis signaling and integrin signaling (Figure 5).

### Confirmation of differentially expressed proteins by western blotting

**Figure 6:**
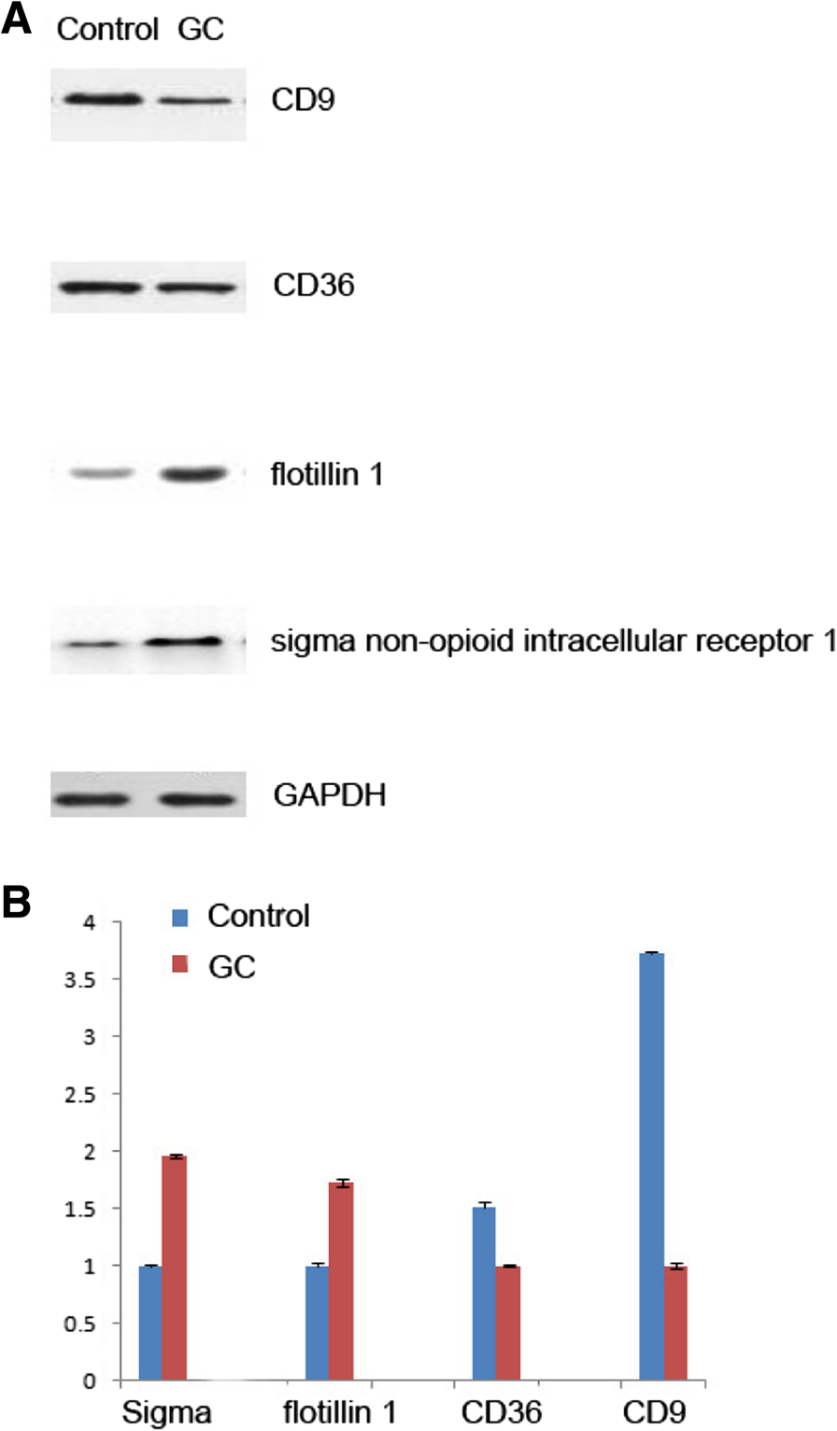
A representative western blot analysis from one of the pools to validate results from TMT labeling. **(A)** Plasma membrane proteins of GC and control tissues were analyzed by Western blot using antibodies against sigma non-opioid intracellular receptor 1, flotillin 1, CD 36 and CD9. **(B)** The levels of non-opioid intracellular receptor 1, flotillin 1, CD 36 and CD 9 were normalized relative to GAPDH levels. Data represent mean values ± SEM.

Western blot analyses were performed on selected candidates (sigma non-opioid intracellular receptor 1, flotillin 1, CD 36 and CD9 molecule). These candidates were chosen based on the plasma membrane markers not known previously reported to be differentially expressed in gastric cancer since the key objective of this study is to identify potential biomarkers of GC. Figure 6 shows that the up- or down-regulation trend of candidate proteins between GC and normal tissue revealed by the Western blot data is congruent with that revealed by quantitative proteomic method. A positive correlation for the direction of changes was observed. The result of western blotting provides evidence that the TMT labeling method for the large scale protein quantification was reliable.

### Flotillin 1 is relevant to clinical gastric cancer as a potential target

**Figure 7:**
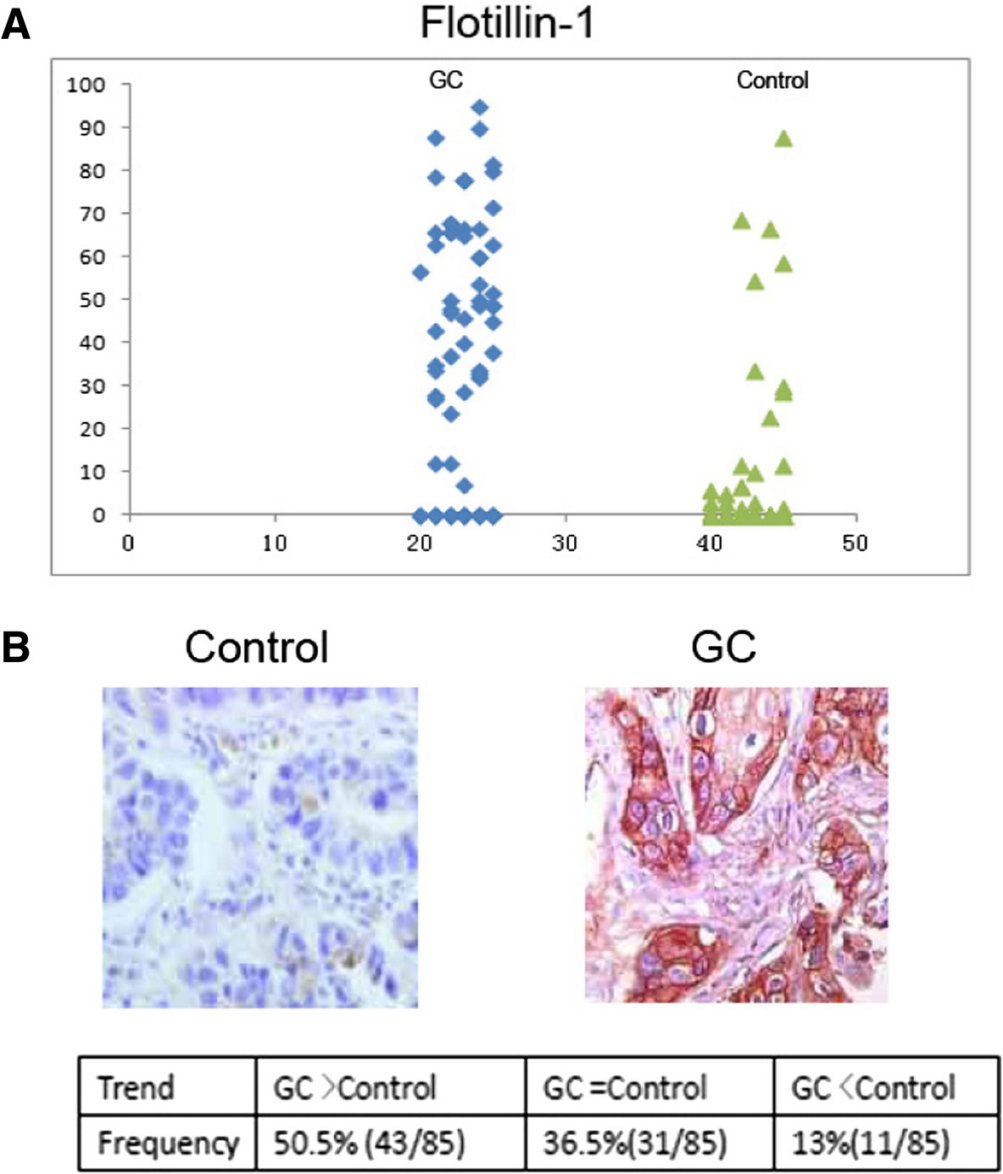
Immunohistochemistry of flotillin 1 in tissue microarrays of clinical gastric samples. A total of 85 matched normal and cancer tissues plus addition 10 normal tissues were analyzed. **(A)** Distribution plot of the IHC scores of flotillin 1 in individual normal and gastric cancer samples. **(B)** Representative IHC images (10× magnification) of flotillin 1 in 2 matched gastric cancer and normal tissues.

To assess the clinical relevance, we examined the expression of flotillin 1 in tissue microarrays containing 85 matched normal and gastric cancer tissues by immunohistochemistry (Additional file 3: Table S3). The TMA also includes ten additional unmatched normal gastric tissues. The expression levels of flotillin 1 across the clinical samples are presented in a distribution plot (Figure 7). Two-samples t test revealed that the expression of flotillin 1 in cancer/tumor samples is significantly higher than that of noncancer/normal tissues (p < 0.01). In addition, 50.5% (43/85) of the matched cases showed higher flotillin 1 expression in the tumor compared to normal tissues while only 13% of the matched cases showed the reverse trend (Figure 7). 36.5% of the matched cases had no detectable level of flotillin 1. The expression data from clinical samples analysis revealed that the upregulation of flotillin 1 has quite a high penetrance (>40%) in gastric cancer. Representative images of the immunohistochemistry of flotillin 1 in 2 sets of matched normal and gastric cancer tissues are shown in Figure 7.

## Discussion

Although the prevalence of gastric cancer is declining and varying geographically, it remains one of the most common cancers in worldwide [1,2,23]. Five-year survival rates have ranged from 90% to less than 5%, mainly depending on the stage of diagnosis [24]. If gastric cancer can be detected and treated in early stages(stage I), the five-year survival rate is better than 90%. Unfortunately, no reliable diagnostic biomarkers exist for early detection of gastric cancer [25]. In order to dig out new drug targets or biomarkers, methods such as subcellular proteome research were adopted to offer new insights [26]. Because most of the drug targets are proteins located in the plasma membrane, we specifically focused our study on the plasma membrane proteome [27]. In this research, we used a percoll/sucrose density gradient approach for plasma membrane enrichment combined with TMT technology using nano liquid chromatography–tandem mass spectrometry analysis to identify specifically differentially expressed proteins in GC tissues compared with adjacent normal tissues. Based on the stringent criteria, in the present study, 82 plasma membrane proteins were identified as differentially expressed proteins in GC, of which 58 proteins were increased and 24 were decreased. Discs, large homolog 1 protein is the most decreased protein and transmembrane 4 L protein is the most increased protein in GC tissue. Some low-abundance plasma membrane proteins such as potassium channel tetramerization domain containing 12, sigma non-opioid intracellular receptor 1 were found.

Previously studies have found a number of markers (e.g. annexin A6, caveolin 1, epidermal growth factor receptor, integrin beta 4) measured in the first and early second trimesters which are associated with the diagnosis of GC [28-35]. Annexin A6 functions as a tumor suppressor in gastric cancer cells through the inhibition of Ras/MAPK signaling [36]. Caveolin-1 promotes gastric cancer progression by up-regulating epithelial to mesenchymal transition by crosstalk of signalling mechanisms under hypoxic condition. Human epidermal growth factor receptor (EGFR) which belongs to EGFR family is overexpressed in a significant proportion of cases of GC to promote metastasis of cancer [37]. Integrin beta 4 expressions are positively correlated in gastric cancer cell lines and tissues [33]. The survival analyses show that the expression of integrin beta 4 is associated with poor outcomes in gastric cancer patients [38]. Our prospective studies have confirmed these validated biomarkers. In addition, many potentially novel biomarkers of GC were found, such as CD36,CD81,CD9,CD97 and so on. As demonstrated here, TMT labeling combined with LC-MS/MS is a powerful tool for the identification of membrane protein biomarkers of GC.

It is reported that endocytosis is enhanced and skewed in cancer cells [39]. Endocytosis is multicomponent process which entails selective packaging of cell-surface proteins, such as receptors for cytokines and adhesion components, in cytoplasmic vesicles (endosomes). The series of sorting events that determines the fate of internalized proteins, either degradation in lysosomes or recycling back to the plasma membrane. Many proteins involved in endocytosis have been reported to be perturbed in human cancers [40]. In this research, proteins such as caveolin 1, epidermal growth factor receptor, major histocompatibility complex, integrin, cortactin, transferrin receptor, ubiquitin specific peptidase 9 participated in caveolae /clathrin mediated endocytosis were found as differentially expressed proteins. Caveolae, subsarcolemmal membrane compartments, have been implicated in signal transduction and vesicular trafficking [41]. Caveolae are capable of removing proteins from the plasma membrane by sequestration and endocytotic mechanism [42]. Clathrin-mediated endocytosis is the endocytic portal into cells through which cargo is packaged into vesicles with the aid of a clathrin coat [43]. Clathrin heavy chain and cortactin have been reported to have their expression levels changed in breast cancer [44,45]. These two proteins were also found overexpressed in GC tissues in this research. However, proteins such as flotillin 1 involved in clathrin-mediated endocytosis have not been reported to differentially expressed in GC tumours in previous researches. The flotillin protein family has been demonstrated to be involved in the development and progression of various cancers. Flotillin 1 is highly conserved protein that localize into specific cholesterol rich microdomains in cellular membranes [46]. Flotillin-2 is a major protein from caveolae/lipid raft and is involved in epidermal cell adhesion. Recent findings have revealed that flotillin1 and flotillin 2 are frequently overexpressed in various types of human cancers including lung adenocarcinoma, nasopharyngeal carcinoma [47], esophageal squamous cell carcinoma, breast cancer and hepatocellular carcinoma [48]. Importantly, recent researches have suggested that the flotillin 2 protein expression is significantly correlated with cancer progression and poor prognosis in gastric carcinomas, probably due to its role in the regulation of cell proliferation, migration, and invasion in gastric carcinoma cells [49,50]. However, flotillin 2 was not identified in our research. After analysis of spotted sequences, we found the peptide similarities of flotillin 1 and 2 were low, as described in supplemental Figure 1(Additional file 4: Figure S1). In this research, abnormal expression of flotillin 1 has been also confirmed in clinical gastric cancers in this study. For the first time, the association of flotillin 1 in gastric cancer has been established in our study, suggesting that flotillin 1 is a promising candidate for future biomarker development for gastric cancer.

## Conclusion

With the help of proteomics analysis, we discovered that a series of plasma membrane proteins showed an altered expression level in GC tissues. 82 plasma membrane proteins with functional relevance to GC were found to be significantly different between GC and control tissues. Our approach allowed us to identify a number of markers (e.g. annexin A6, caveolin 1 ,epidermal growth factor receptor, integrin beta 4) that were previously reported as biomarkers of GC. Additionally, we have presented several potential biomarkers participated in endocytosis pathway and integrin signaling pathway were firstly identified differentially expressed in GC samples. Our findings also suggest that flotillin 1 may be a novel biomarker for GC.These findings will not only benefit early diagnosis of this cancer at the molecular level but also improve our understanding of the initiation and development of GC.

## Additional files

Additional file 1: Table S1. All of identified proteins in this research. Additional file 2: Table S2. The list of the differentially expressed proteins in GC samples. Additional file 3: Table S3. Raw IHC scores of climnical samples used in TMAS. Additional file 4: Figure S1. CLUSTAL 2. 1 multiple sequence alignment.
