# Supplementary figures and images for: Plasma membrane proteomic analysis of human Gastric Cancer tissues: revealing flotillin 1 as a marker for Gastric Cancer
Source: BMC Cancer. 2015 May 7;15:367. doi: 10.1186/s12885-015-1343-5 (PMC4525731; doi:10.1186/s12885-015-1343-5)

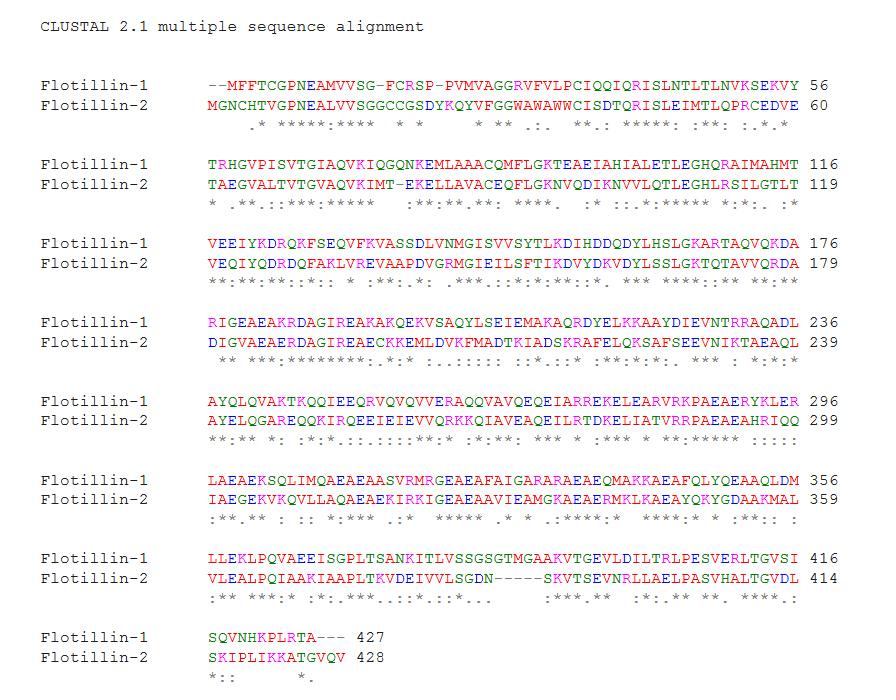

Supplement: Additional file 4: Figure S1. — CLUSTAL 2. 1 multiple sequence alignment. [file 12885_2015_1343_MOESM4_ESM.jpeg]
